# Effects of extruded flaxseed and Salmate^®^ inclusion in the diet on milk yield and composition, ruminal fermentation and degradation, and kinetic flow of digesta and fluid in lactating dairy cows in the subtropics

**DOI:** 10.14202/vetworld.2024.540-549

**Published:** 2024-03-07

**Authors:** Mohammed Al-Saiady, Tarek Al-Shaheen, Ahmed El-Waziry, Abd El-Nasser Ahmed Mohammed

**Affiliations:** 1 Department of ARASCO Research and Development, P.O. Box 53845, Riyadh, 11593, Kingdom of Saudi Arabia; 2 Department of Animal and Fish Production, College of Agricultural and Food Sciences, King Faisal University, P.O. Box 420, Al-Hassa, 31982, Kingdom of Saudi Arabia; 3 Department of Animal and Fish Production, Faculty of Agriculture, El-Shatby, Alexandria University, P.O. Box 21454, Egypt

**Keywords:** degradability, flaxseed, passage rate, Salmate^®^ ruminal fermentation

## Abstract

**Background and Aim:**

Dietary supplements play pivotal roles in promoting productive and reproductive performance in ruminant animals. The aims of the present study were to evaluate the effects of extruded flaxseed and Salmate^®^ (Ballard Group, Inc, OH, USA) inclusion in diets on milk yield and composition, ruminal degradation and fermentation, and flow of fluids and digesta in lactating cattle.

**Materials and Methods:**

Six rumen-fistulated Holstein lactating cows were distributed to a 6 × 6 design of Latin square (L.S.). The groups were assorted into a control group fed a basal control diet and two treated groups fed diets containing extruded flaxseed (7.0%) or Salmate^®^ (25 g/head/day). The basal control, extruded flaxseed, and Salmate^®^ diets were formulated as isonitrogenous and isoenergetic. Each L.S. period of the group comprised 21 days, including 10 days for adaptation to the diet and 11 days for data sampling and recording.

**Results:**

Feed intake did not differ among the control, extruded flaxseed, and Salmate^®^ groups. Milk yield (kg) and protein and fat composition (%) were improved on feeding the extruded flaxseed diet compared with the Salmate^®^ and control diets. Extruded flaxseed or Salmate^®^ diet had no effect on the values of ruminal pH, ammonia, and volatile fatty acids except isobutyrate, which decreased in the Salmate^®^ group. Degradable efficiency and ruminal digestibility were significantly decreased with the inclusion of extruded flaxseed and/or Salmate^®^ in the diets. The extruded flaxseed and Salmate^®^ groups had a greater digesta passage rate than the control group. The extruded flaxseed and control groups had a greater liquid passage rate than the Salmate^®^ group.

**Conclusion:**

The inclusion of extruded flaxseed in the diet improved (p < 0.05) milk yield, milk composition, and milk Omega-6: Omega-3 ratio with no changes in ruminal fermentation, notable negative effects on degradable efficiency and ruminal digestibility.

## Introduction

Dietary supplements to mammals affect productive and reproductive performance [1]. Mammalian species can synthesize all of the fatty acids (FAs) with the exception of omega-3 and omega-6 families, which should be supplied in the diets. The problems with the supplementation of essential FAs to ruminant animals are the occurrence of lipolysis and biohydrogenation of FAs in the rumen. Very few unsaturated FAs are available for absorption, and the toxic effect of unsaturated FAs on rumen microbes is indicated [2]. Ruminal biohydrogenation results in 70%–90% saturation of dietary unsaturated FAs [3]. Therefore, the pathway of rumen biohydrogenation requires fat manipulation to ensure that dietary unsaturated FAs reach the small intestine in the form of conjugated linoleic acid. This can be achieved through the ingredients of feed [4, 5], shifting the ability of rumen bypass [6], and changing the environment of the rumen [7].

Different factors modify milk yield and composition [8–10]. Feeding strategies can be used to modify milk yield and composition as lipid supplementation [11, 12]. Rumen-protected fats from different sources have been used to improve physiological responses, milk yield, and composition in ruminants [10, 13]. Protecting fat from ruminal biohydrogenation determines the efficiency of FAs transferred into milk and the percentage of n-3 FA improvements in milk [4]. Several studies have evaluated the effects of fat supplementation on milk yield and composition [14, 15]. However, caution should be taken with regard to dietary fat supplementation due to the significant decrease in feed intake [16]. In addition, saturated free FAs (FFAs) appeared to induce insulin resistance and increase the amount of glucose required to synthesize lactose and, consequently, for milk yield [17]. Supplementing 200 g/day of fish oil resulted in a 26% reduction in milk fat yield compared with 200 g/day of olive oil [18]. Therefore, the objective is to supply essential FAs without compromising rumen production. Moreover, non-optimal environmental conditions, including temperature (°C) and relative humidity (%), are well known to compromise the productive and reproductive performances of ruminant animals in the subtropics [19, 20]. Production efficiency is negatively affected when the ambient temperature is above or below threshold values.

Therefore, the aims of this study were to explore the effect of the inclusion of extruded flaxseed and Salmate^®^ (Ballard Group, Inc, OH, USA) as dietary omega-3 sources on (1) feed intake, milk yield and composition, and milk FA profiles; (2) ruminal fermentation and degradation; and (3) kinetic flow of digesta and fluid under prevailing hot summer conditions in Riyadh, Saudi Arabia.

## Materials and Methods

### Ethical approval

All experimental procedures performed for animal care in this study were approved according to the scientific research deanship ethical standards of King Faisal University (Approval No. EA005854).

### Study period and location

The study was conducted from June to December 2023 in Arasco commercial dairy farm located in South Riyadh, Kingdom of Saudi Arabia. The range of ambient temperature (°C) was 31.0°C–48.0°C and relative humidity (%) was 40.0%–74.0%.

### Animal management and formulation of diets

Six multiparous lactating Holstein cows of the sixth lactation stage were selected for the experiment. The selected lactating cows had 582.0 ± 21.0 kg body weight (BW) and 3.35 ± 0.10 of body condition score. They were surgically equipped to fit rumen cannula. The selected lactating cows were classified using a Latin square design (L.S.) to investigate the effects of extruded flaxseed (7.0%) and Salmate^®^ (25 g/head/day) compared with the control diet. Each L.S. period of the group comprised 21 days, including 10 days for adaptation to the diet and 11 days for data sampling and recording. The extruded flaxseed, Salmate^®^, and control rations (Table-1) were formulated to provide the nutrient requirements of dairy cows according to BW (580.0 kg) and body gain (0.2 kg/day), milk yield (17 kg/day), and milk fat (3.5%) as recommended by the National Research Council [21]. The dairy cows were given a mineral mixture and fresh water *ad libitum*.

**Table-1:** Ingredients and chemical composition of the dietary treatments of lactating Holstein cows.

Parameter	Control	Extruded flaxseed	Salmate^®^
Ingredients, DM, %			
Alfalfa hay	27.96	27.96	27.96
Wheat straw	2.46	2.46	2.46
Soybean meal	3.29	3.22	3.79
Cotton seed	6.66	5.00	6.59
Corn flakes	29.15	26.70	28.67
Buffer	1.46	1.46	1.46
Arasco super soya	3.00	3.00	3.00
Bypass soya artat	2.96	1.97	2.96
Earley high premix	2.69	2.69	2.69
Wheat bran	1.66	1.66	1.66
Soybean hulls	1.33	1.33	1.33
Energizer RP 10*	1.83	---	1.79
Corn silage	11.11	11.11	11.11
Wet corn fiber	4.44	4.44	4.44
Salmate^®^	---	---	**
Flaxseed	---	7.00	---
	100	100	100
Chemical analysis, %			
DM	97.33	97.73	97.74
OM	90.09	90.46	90.56
CP	18.20	18.56	18.79
CF	16.34	16.62	16.40
Ether extract	4.53	5.26	4.98
Nitrogen free extract	50.97	50.02	50.39
Ash	9.96	9.74	9.44
ADF	22.82	19.18	20.35
NDF	38.08	36.61	39.31
Net energy, Mcal/kg DM	1.75	1.75	1.75

Control group fed basal diet. Extruded flaxseed group fed isoenergetic and isonitrogenous basal diet containing supplemented with 7% extruded flaxseed. Salmate^®^ group fed isoenergetic and isonitrogenous basal diet containing 25 g/head/day of dry protected fish oil (**).

* Energizer RP 10, rumen protected fat containing 99% fatty acids. DM=Dry matter, OM=Organic matter, CP=Crude protein, CF=Crude fiber, ADF=Acid detergent fiber, NDF=Neutral detergent fiber

### Feed intake, sample collection, and chemical analyses

The extruded flaxseed and Salmate^®^ rations were prepared weekly to avoid lipid peroxidation. The lactating cows were offered sufficient amounts of extruded flaxseed, Salmate^®^, and control rations as a total mixed ration at 6:0 AM and 4:0 PM to allow for a 10.0% ort. Feed intake was recorded daily from day 16.0^th^ to day 21.0^st^ of each L.S. period as the difference between the daily quantity of ration that was offered and its respective ort. Samples of each extruded flaxseed, Salmate^®^, and control rations and orts were collected daily from cows and stored (−10.0°C). The extruded flaxseed, Salmate^®^, and control samples at the end of each experiment were thawed, pre-dried (65.0°C for 24:0 h), ground and milled (1.0 mm), and dried at 105.0°C for 3.0 h. Chemical analysis (%) of crude fiber (CF), crude protein (CP), ether extract (EE), and ash was performed according to the procedures of Association of Official Analytical Chemists [22]. Acid and neutral detergent fibers were determined according to the procedures of Van Soest *et al*. [23].

### Milk sample collection and chemical analysis

Milk yield and other information of the extruded flaxseed, Salmate^®^, and control groups were recorded electronically in the milking parlor twice a day (06:00 h and 15:00 h) from the 16^th^ to the 21^st^ day of each experimental period. Milk samples (50.0 mL) were collected from the experimental and control groups and stored in tubes for further chemical analysis. Milk samples from the experimental and control groups were analyzed for determination of casein, fat, protein, total solids, solids non-fat, lactose, and milk urea nitrogen. Fat-corrected milk (3.5% fat) and energy-corrected milk (3.5% fat and 3.2% protein) were calculated according to the formulas of Nordlund [24] and Bernard [25]. FA contents were calculated as described by Glasser *et al*. [26]. In addition, individual milk samples were collected on the 17^th^ day of each L.S. period and stored (−20°C) without preservatives to determine FA profiles using gas chromatography [27].

### Ruminal fermentation and degradation

Ruminal parameters were assessed during the first 2 days of each extruded flaxseed, Salmate^®^, and control period [28]. A data logger was placed in the rumen for pH and temperature recording (Dascor Inc, USA) at 3, 6, 9, 12, 24, and 48 h after providing extruded flaxseed, Salmate^®^, and control diets. Samples of ruminal fluid were collected at the aforementioned times of providing the diets and filtered for determination of NH_3_-N, total volatile FAs (VFAs), acetate, propionate, butyrate, isobutyrate, valerate, and isovalerate [29, 30].

Two polyester bags (Swiss nylon monofilament) were used for rumen degradability measurements. Three grams of ground extruded flaxseed, Salmate^®^, and control diets (2 mm) were placed in each bag and inserted into the ventral sac of the rumen at 07:0 am immediately before the morning feeding of each cow. The bags were placed in the rumen and removed later at 3, 6, 12, 24, and 48 h. The bags were then rinsed and manipulated in cold water until the water became clear, squeezed, and stored at –20°C. Ruminal dry matter (DM), organic matter (OM), CP, neutral detergent fiber (NDF), and acid detergent fiber (ADF) degradation was measured using the nylon bag technique. The DM, OM, CP, NDF, and ADF disappearances were determined by fitting the individual values to the equation: P = a + b (1-exp^-ct^), where P is the disappearance rate after time t (h), and a, b, and c are the least squares estimated for the soluble fraction, degradable fraction, and the rate of degradation, respectively.

### Solid and liquid passage rates

Solid passage rates for extruded flaxseed, Salmate^®^, and control cows were estimated using chromium oxide (Cr_2_O_3_) [31]. Each cow was administered 14 g of Cr_2_O_3_ in a single dose through a ruminal fistula. Fecal samples were collected directly from the rectum after dosing with Cr_2_O_3_ dose. Fecal samples were dried in an oven at 55.0°C (72:0 h), milled (1.0 mm), and stored for analysis of Cr_2_O_3_ content using spectrophotometry at 400:0 μm and calculated from a standard curve [32].

Liquid passage rates for extruded flaxseed, Salmate^®^, and control cows were estimated using polyethylene glycol. Each cow was administered 100 g of polyethylene glycol through a ruminal fistula (diluted in 200 mL of distilled water). Polyethylene glycol concentrations in the samples were determined in ruminal fluids using gas chromatography.

### Statistical analysis

Data of milk, ruminal fermentation, and degradability were analyzed by the generalized linear model procedure of SAS [33] using a factorial experiment following an L.S. design where cow, time, and diet were considered the main effects according to the following model: Y_ijkl_ = μ + C_i_ + T_j_ + D_k_ + T_j_*D_k_ + e_ijkl_; where: Y_ijkl_: The observation, μ: The overall mean, C_i_ is the effect of cow, T_j_ is the effect of time, D_k_ is the effect of diet, T_j_*D_k_ is the interaction between time and diet, and e_ij_: Standard error.

## Results

### Chemical composition of the diets

The chemical composition was similar among the extruded flaxseed, Salmate^®^, and control diets (Table-1). In addition, the diets of extruded flaxseed, Salmate^®^, and control were approximately isonitrogenous (CP %; 18.35 vs. 18.46 vs. 18.79) and isoenergetic (net energy, 1.75 Mcal/kg DM). The contents of the extruded flaxseed diet were lower than those of the Salmate^®^ and control diets in terms of the percentage of soybeans, cotton seed, corn flakes, and bypass soya arhat. CF values (%) were lower in the control diet than in Salmate^®^ and extruded flaxseed (6.34 vs. 6.90 and 9.02, respectively). In addition, EE values (%) were lower in the control diet than in the Salmate^®^ and 7 (4.53 vs. 4.98 and 6.29, respectively).

### Feed intake, milk yield, and composition

The results of feed intake, milk yield, and composition are presented in Table-2. Feed intake did not differ (p > 0.05) between the extruded flaxseed and Salmate^®^ groups compared with the control group. Cows fed the extruded flaxseed diet produced more milk (p < 0.05) than those fed the Salmate^®^ and control diets, whereas milk yield efficiency did not differ (p > 0.05) among groups. Milk values of casein (%), density (g/cm^3^), and protein (%) were significantly improved in the extruded flaxseed group compared with the control and/or Salmate^®^ groups. In addition, milk values of fat, solid not fat, and FFAs (%) of the extruded flaxseed group were decreased (p < 0.05) compared with those of the Salmate^®^ and control groups. Percentages of omega-6 and omega-3 FAs in the milk of extruded flaxseed were significantly higher than those of the control and Salmate^®^ groups. In addition, the omega-6: omega-3 ratio decreased from (8.06) in the control group and (8.31) in the Salmate^®^ group to (3.76) in the extruded flaxseed group (p < 0.05).

**Table-2:** Effects of extruded flaxseed and salmate^®^ supplementation in the diet of lactating Holstein cows on feed intake and milk yield and composition.

Parameter	Control	Extruded flaxseed	Salmate^®^
Feed intake, kg	30.02 ± 0.54	29.26 ± 0.54	28.98 ± 0.54
Milk yield, kg	16.85^b^ ± 0.34	17.65^a^ ± 0.34	16.59^b^ ± 0.34
Production efficiency, kg milk/kg feed	0.56 ± 0.06	0.60 ± 0.06	0.57 ± 0.06
Casein, %	2.32^a^ ± 0.01	2.30^a^ ± 0.01	2.17^b^ ± 0.01
Density g/cm^3^	1028.46^b^ ± 0.12	1029.81^a^ ± 0.13	1028.35^b^ ± 0.12
Protein, %	2.85^b^ ± 0.01	2.94^a^ ± 0.01	2.74^c^ ± 0.01
Fat, %	3.68^a^ ± 0.05	3.04^c^ ± 0.06	3.38^b^ ± 0.05
Total solid, %	12.20 ± 0.73	11.17 ± 0.78	12.67 ± 0.70
Solid not fat, %	8.52^b^ ± 0.02	8.13^c^ ± 0.02	9.29^a^ ± 0.02
Lactose, %	4.40 ± 0.01	4.42 ± 0.01	4.39 ± 0.01
Urea, %	0.03 ± 0.001	0.03 ± 0.001	0.04 ± 0.001
FFA, %	4.49^a^ ± 0.11	2.52^b^ ± 0.12	4.29^a^ ± 0.10
C_18_H_32_O_2_ (Omega-6)	2.66^b^ ± 0.03	2.86^a^ ± 0.03	2.66^b^ ± 0.03
C_18_H_30_O_2_ (Omega-3)	0.33^b^ ± 0.01	0.76^a^ ± 0.01	0.32^b^ ± 0.01
Omega-6: Omega-3	8:1	~ 4:1	8:1

^a,b,c^Values in the same row with different superscripts differ significantly (p < 0.05). Control group fed basal diet. Extruded flaxseed group fed isoenergetic and isonitrogenous diet containing supplemented with 7% extruded flaxseed. Salmate^®^ group fed isoenergetic and isonitrogenous diet containing 25 g/head/day of dry protected fish oil. FFAs=Free fatty acids

### Ruminal disappearance and degradability characteristics

The ruminal degradability level (%) of DM, OM, CP, and acid and neutral detergent fibers was determined at 3, 6, 9, 12, 24, and 48 h after feeding control, extruded flaxseed, and Salmate^®^ diets (Table-3). The results showed that DM, OM, CP, and acid and neutral detergent fiber degradability (%) were lower (p < 0.05) in the extruded flaxseed and Salmate^®^ groups than in the control group. Acid and neutral detergent fibers’ degradability was significantly (p < 0.05) the lowest of the extruded flaxseed and Salmate^®^ groups compared with the control. The degradation level of neutral detergent fiber was the lowest in the extruded flaxseed group and the highest in the Salmate^®^ group compared with the control group.

**Table-3:** Effects of extruded flaxseed and salmate^®^ supplementation in the diet of lactating Holstein cows on ruminal degradability level per time (%) of DM, OM, CP, ADF and NDF.

Parameter	Incubation time, h	Control	Extruded flaxseed	Salmate^®^
DM	3	21.41 ± 2.53	16.87 ± 2.92	21.44 ± 2.53
	6	26.60 ± 2.53	21.72 ± 2.92	26.71 ± 2.53
	9	37.10^a^ ± 2.53	24.68^c^ ± 2.92	31.78^b^ ± 2.53
	12	39.15^a^ ± 2.53	32.15^b^ ± 2.92	34.64^b^ ± 2.53
	24	49.43^a^ ± 2.53	38.92^b^ ± 2.92	46.33^a^ ± 2.53
	48	64.54^a^ ± 2.53	53.29^b^ ± 2.92	55.38^b^ ± 2.53
OM	3	19.38 ± 2.53	13.99 ± 2.53	19.78 ± 2.53
	6	23.98 ± 2.53	18.70 ± 2.53	24.63 ± 2.53
	9	34.39^a^ ± 2.53	22.90^b^ ± 2.53	29.65^a^ ± 2.53
	12	36.64 ± 2.53	31.61 ± 2.53	32.40 ± 2.53
	24	47.54^a^ ± 2.53	42.78^a^ ± 2.53	40.65^b^ ± 2.53
	48	63.57^a^ ± 2.53	54.32^b^ ± 2.53	54.38^b^ ± 2.53
CP	3	21.85 ± 2.77	18.95 ± 2.64	19.96 ± 2.92
	6	23.27 ± 2.77	20.90 ± 2.64	20.66 ± 2.92
	9	28.81 ± 2.77	23.05 ± 2.64	22.75 ± 2.92
	12	31.09 ± 2.77	26.42 ± 2.64	25.59 ± 2.92
	24	39.09 ± 2.77	33.43 ± 2.64	33.68 ± 2.92
	48	58.19^a^ ± 2.77	42.75^b^ ± 2.64	39.28^b^ ± 2.92
ADF	3	16.08^a^ ± 3.58	4.34^b^ ± 2.64	12.36^a^ ± 3.32
	6	14.82^a^ ± 3.58	4.75^b^ ± 2.64	15.00^a^ ± 3.32
	9	16.98^a^ ± 3.58	4.85^b^ ± 2.64	15.47^a^ ± 3.32
	12	16.12^a^ ± 3.58	7.87^b^ ± 2.64	16.41^a^ ± 3.32
	24	30.92^a^ ± 3.58	14.06^b^ ± 2.64	24.24^a^ ± 3.32
	48	52.84^a^ ± 3.58	26.92^b^ ± 2.64	27.03^b^ ± 3.32
NDF	3	23.13^b^ ± 2.77	22.07^b^ ± 2.77	30.83^a^ ± 2.77
	6	24.29^b^ ± 2.77	22.95^b^ ± 2.77	31.11^a^ ± 2.77
	9	28.44^a^ ± 2.77	22.96^b^ ± 2.77	32.14^a^ ± 2.77
	12	28.95^a^ ± 2.77	23.06^b^ ± 2.77	32.93^a^ ± 2.77
	24	39.45 ± 2.77	34.46 ± 2.77	41.09 ± 2.77
	48	50.38^a^ ± 2.77	43.90^b^ ± 2.77	51.83^a^ ± 2.77

^a,b,c^Values in the same row with different superscripts differ significantly (p < 0.05). Control group fed basal diet. Extruded flaxseed group fed isoenergetic and isonitrogenous diet containing supplemented with 7% extruded flaxseed. Salmate^®^ group fed isoenergetic and isonitrogenous diet containing 25 g/head/day of dry protected fish oil. DM=Dry matter, OM=Organic matter, CP=Crude protein, ADF=Acid detergent fiber, NDF=Neutral detergent fiber

Degradability characteristics and effective degradability values of DM, OM, CP, ADF, and NDF as soluble fraction (*a*), degradable fraction (*b*), degradation rate (*c*), undegradable fraction (*ud*), and effective degradability (*ED*) of extruded flaxseed and Salmate^®^ diets are presented in Table-4. The effective degradability of DM, OM, CP, ADF, and NDF was the lowest in the extruded flaxseed group, followed by the Salmate^®^ and control groups, except for the effective degradability of NDF in the extruded flaxseed group, which was the highest (Table-5). In addition, *“b*” of NDF was the highest in the extruded flaxseed group compared with the control and Salmate^®^ groups.

**Table-4:** Effects of extruded flaxseed and Salmate^®^ supplementation in the diet of lactating Holstein cows on degradability characteristics and effective degradability values of DM, OM, CP, ADF, and NDF.

Parameter	Control	Extruded flaxseed	Salmate^®^
DM, %			
Soluble fraction, *a*	13.21 ± 2.15	12.17 ± 2.63	11.02 ± 2.24
Degradable fraction, *b*	53.81 ± 8.14	46.53 ± 9.97	51.21 ± 8.50
Rate of degradation, *c*	0.055 ± 0.01	0.047 ± 0.01	0.071 ± 0.01
Undegradable fraction, *ud*	32.97 ± 7.95	41.29 ± 9.73	37.76 ± 8.30
Effective degradability, *ED*	41.08^a^ ± 1.59	33.58^b^ ± 1.95	36.95^b^ ± 1.66
OM, %			
Soluble fraction, *a*	12.26 ± 2.24	12.00 ± 2.81	14.98 ± 2.24
Degradable fraction, *b*	55.08 ± 8.50	48.69 ± 10.66	46.14 ± 8.50
Rate of degradation, *c*	0.046 ± 0.01	0.041 ± 0.01	0.042 ± 0.01
Undegradable fraction, *ud*	32.65 ± 8.30	39.29 ± 10.41	38.87 ± 8.30
Effective degradability, *ED*	39.23^a^ ± 1.66	33.28^b^ ± 2.09	35.02^b^ ± 1.66
CP, %			
Soluble fraction, *a*	15.12 ± 2.48	15.38 ± 2.35	15.87 ± 2.63
Degradable fraction, *b*	60.06 ± 9.40	39.65 ± 8.92	41.08 ± 9.97
Rate of degradation, *c*	0.035 ± 0.01	0.035 ± 0.01	0.038 ± 0.01
Undegradable fraction, *ud*	24.80 ± 9.18	44.96 ± 8.71	43.04 ± 9.73
Effective degradability, *ED*	35.34^a^ ± 1.84	27.42^b^ ± 1.74	27.92^b^ ± 1.95
ADF, %			
Soluble fraction, *a*	7.58^a^ ± 3.04	2.65^b^ ± 3.04	8.69^a^ ± 3.04
Degradable fraction, *b*	68.37^a^ ± 11.51	23.53^b^ ± 11.51	56.07^a^ ± 11.51
Rate of degradation, *c*	0.023^b^ ± 0.01	0.003^b^ ± 0.01	0.061^a^ ± 0.01
Undegradable fraction, *ud*	24.03^c^ ± 11.24	26.18^b^ ± 11.24	35.22^a^ ± 11.24
Effective degradability, *ED*	25.86^a^ ± 2.25	10.51^c^ ± 2.25	18.92^b^ ± 2.25
NDF, %			
Soluble fraction, *a*	17.71^b^ ± 2.35	16.30^b^ ± 2.35	25.72^a^ ± 2.35
Degradable fraction, *b*	48.24 ± 8.92	53.14 ± 8.92	41.95 ± 8.92
Rate of degradation, *c*	0.034 ± 0.01	0.029 ± 0.01	0.047 ± 0.01
Undegradable fraction, *ud*	34.04 ± 8.71	30.55 ± 8.71	32.31 ± 8.71
Effective degradability, *ED*	33.57^a^ ± 1.74	28.89^b^ ± 1.74	36.86^a^ ± 1.74

^a,b,c^Values in the same row with different superscripts differ significantly (p < 0.05). Control group fed basal diet. Extruded flaxseed group fed isoenergetic and isonitrogenous diet containing supplemented with 7% extruded flaxseed. Salmate^®^ group fed isoenergetic and isonitrogenous diet containing 25 g/head/day of dry protected fish oil. DM=Dry matter, OM=Organic matter, CP=Crude protein, ADF=Acid detergent fiber, NDF=Neutral detergent fiber

**Table-5:** Effects of extruded flaxseed and Salmate^®^ supplementation in the diet of lactating Holstein cows on ruminal pH, temperature, ammonia concentrations (NH3-N), and VFAs concentrations in ruminal fluid.

Parameter	Treatments		

	Control	Extruded flaxseed	Salmate^®^
pH	6.26 ± 0.004	6.21 ± 0.006	6.24 ± 0.006
Temperature, °C	39.08^a^ ± 0.02	38.94^b^ ± 0.02	39.12^a^ ± 0.02
NH_3_-N, mg/dL	11.48 ± 1.13	10.19 ± 1.51	11.22 ± 1.23
Total VFAs, mM	113.43 ± 9.25	130.81 ± 9.25	114.55 ± 9.25
Acetate, % molar	50.71 ± 1.94	51.71 ± 1.94	49.04 ± 1.94
Propionate, % molar	30.08 ± 2.61	29.50 ± 2.61	35.04 ± 2.61
Butyrate, % molar	9.43 ± 0.67	8.71 ± 0.67	7.82 ± 0.67
Isobutyrate, % molar	7.32^a^ ± 0.70	7.70^a^ ± 0.70	5.69^b^ ± 0.70
Valerate, % molar	1.28 ± 0.14	1.19 ± 0.14	1.14 ± 0.14
Isovalerate, % molar	1.15 ± 0.28	1.16 ± 0.28	1.24 ± 0.28
Acetate: propionate ratio	1.68	1.75	1.39

^a,b,c^Values in the same row with different superscripts differ significantly (p < 0.05). Control group fed basal diet. Extruded flaxseed group fed isoenergetic and isonitrogenous diet containing supplemented with 7% extruded flaxseed. Salmate^®^ group fed isoenergetic and isonitrogenous diet containing 25 g/head/day of dry protected fish oil. VFAs=Volatile fatty acids

### Ruminal fermentation

The effects of extruded flaxseed and Salmate^®^ supplementation in the diet of lactating Holstein cows on ruminal pH, temperature, ammonia, and VFA concentrations in the ruminal fluid are shown in Table-5. There was no change (p > 0.05) in the ruminal pH of either the extruded flaxseed or Salmate^®^ groups compared with the control group. On the other hand, there was a significant decrease (p < 0.05) in the ruminal temperature of the extruded flaxseed group compared with the Salmate^®^ and control groups. The data in Table-5 show that ruminal fermentation as measured by NH_3_-N, total and individual VFAs, acetate, propionate, butyrate, valerate, and isovalerate molar proportions were not affected by the addition of extruded flaxseed and Salmate^®^ to the basal diet. The isobutyrate molar proportion was significantly higher (p < 0.05) in cows fed the extruded flaxseed (7.70) and control (7.32) diets than in those fed the Salmate^®^ diet (5.69).

### Kinetics of the solid and fluid passage rates

The results of the solid (digesta) passage rate using the chromium oxide marker are presented in Table-6. The results show that the regression coefficient values of the extruded flaxseed (−0.013) and Salmate^®^ (−0.012) groups are significantly higher (p < 0.0001) compared with the regression coefficient of the control (−0.007) group, which indicates that the extruded flaxseed and Salmate^®^ diets resulted in a greater solid passage rate than the control basal diet. Thus, increasing the time by 1 h decreased the passage rate of digesta out of the rumen by 0.007 in the control group compared with 0.012 and 0.013 in the Salmate^®^ and extruded flaxseed groups, respectively.

**Table-6:** Effects of extruded flaxseed and Salmate^®^ supplementation in the diet of lactating Holstein cows on solid phase passage rate using chromium oxide marker (14 g/head).

Parameter	Treatments		

	Control	Extruded flaxseed	Salmate^®^
Regression coefficient values	−0.007^a^ ± 0.001	−0.013^b^ ± 0.001	−0.012*^β^* ± 0.001
Total retention time, h=A/B	56.42	40.69	47.41

^a,b^Values in the same row with different superscripts differ significantly (p < 0.05). Control group fed basal diet. Extruded flaxseed group fed isoenergetic and isonitrogenous diet containing supplemented with 7% extruded flaxseed. Salmate^®^ group fed isoenergetic and isonitrogenous diet containing 25 g/head/day of dry protected fish oil

The results of the liquid passage rate using the polyethylene glycol marker are presented in Table-7. The results show that the regression coefficients of the fluid passage rate of the extruded flaxseed (−0.028) and control (−0.021) groups were significantly (p < 0.0001) different from that of the Salmate^®^ group (−0.019), thus indicating that the extruded flaxseed and control diets had greater fluid passage rates than the Salmate^®^ diet. In addition, the fluid passage rate through the regression coefficient of the extruded flaxseed diet was significantly higher than that of the Salmate^®^ diet. The results indicate that increasing the time by 1 h decreases the passage rate of the liquid phase out of the rumen by (0.021) in the control group and (0.019) and (0.028) in the groups of Salmate^®^ and extruded flaxseed, respectively.

**Table-7:** Effects of extruded flaxseed and Salmate^®^ supplementation in the diet of lactating Holstein cows on liquid phase passage rate using Polyethylene glycol maker (100 g/head).

Parameter	Treatments		

	Control	Extruded flaxseed	Salmate^®^
Regression coefficient values	−0.021^b^ ± 0.004	−0.028^a^ ± 0.004	−0.019^c^ ± 0.004
Rumen retention time, h=A/B	16.38	14.03	16.68

^a,b,c^Values in the same row with different superscripts differ significantly (p < 0.05). Control group fed basal diet. Extruded flaxseed group fed isoenergetic and isonitrogenous diet containing supplemented with 7% extruded flaxseed. Salmate^®^ group fed isoenergetic and isonitrogenous diet containing 25 g/head/day of dry protected fish oil

## Discussion

The effects of extruded flaxseed (7.0%) and Salmate^®^ (25 g/head/day) inclusion in the diets compared with the basal control diet, which was fed to lactating cows, on milk yield and composition, ruminal degradation and fermentation, flow of fluids, and digesta are presented in Tables 1-7. The results indicated that feed intake did not differ between the control and treated groups. Values of milk yield (kg), protein, and fat (%) were improved on feeding an extruded flaxseed diet. Extruded flaxseed or Salmate^®^ diet had no effect on ruminal pH, ammonia, or VFA values except isobutyrate, which decreased in the Salmate^®^ group. Degradable efficiency and ruminal digestibility were significantly decreased on the inclusion of extruded flaxseed and/or Salmate^®^ in the diets. The extruded flaxseed and Salmate^®^ groups had a greater digesta passage rate than the control group. The extruded flaxseed and control groups had a greater liquid passage rate than the Salmate^®^ group.

### Feed composition

Chemical compositions were generally similar among extruded flaxseed, Salmate^®^, and control diets (Table-1). EE concentration (%) was lower for the control diet than for the Salmate^®^ and extruded flaxseed diets (4.53 vs. 4.98 and 5.26 %, respectively). Although the extruded flaxseed and Salmate^®^ diets were formulated to have similar values of EE, the increase in dietary EE concentrations of the extruded flaxseed and Salmate^®^ diets was due to lipid contents (Table-1). The greatest difference in the EE content of diets was between extruded flaxseed, control, and Salmate^®^ diets, which must be considered to interpret differences between the treatment and control groups on the measured parameters. In addition, the values of monounsaturated FAs and polyunsaturated FAs (monounsaturated fat and polyunsaturated fat) were the highest and the lowest in the control diet, followed by the Salmate^®^ and extruded flaxseed diets, respectively. Petit [34] indicated that flaxseed is a rich source of cis-9, cis-12, and cis-15 18:3, thus increasing the relative proportion of linolenic acid in the extruded flaxseed and Salmate^®^ diets.

### Feed intake, milk yield, and composition

The values of feed intake, milk yield, and composition are presented in Table-2. Feed intake was decreased (p > 0.05) in the extruded flaxseed and Salmate^®^ groups compared with the control group. It has been suggested that feedback satiety signals may be generated to prevent further feed intake on feeding large numbers of fats [35]. Lactating cows fed an extruded flaxseed diet produced more milk (p < 0.05) than those fed a Salmate^®^ or control diet. This could be attributed to the increase in EE concentration in the extruded flaxseed diet compared with the Salmate^®^ and control diets (5.26 vs. 4.98 and 4.53, respectively), thus indicating more energy spared for milk yield. Milk yield efficiency was increased (p > 0.05) in the extruded flaxseed and Salmate^®^ groups compared with the control group. The results of the present study are in agreement with the findings of Benchaar *et al*. [36], who reported an increase in milk yield due to feeding animals with flax oil in the diet, and they did not report any significant decrease in DM intake. Moreover, feeding flaxseed in other studies [37, 38] did not affect feed intake and positively impacted milk yield. Other investigators reported a reduction in DMI due to the addition of fat to the diet of lactating cows. It is important to mention that a high-fat content in the diet of lactating cows might decrease feed intake and fiber digestibility, leading to the ruminal sensation of fill [39].

There was a significant increase in milk protein (%) in the extruded flaxseed group compared with the control and Salmate^®^ groups (2.94 vs. 2.85 and 2.74; p < 0.05), which might be due to the increase in CP and EE (%) in the extruded flaxseed diet. On the other hand, a decrease in milk protein percentage was shown with Salmate^®^ diet supplementation in this study, as in other studies [40, 41]. A decrease in milk protein (%) during dietary fat inclusion was attributed to a shortage of amino acids available for protein synthesis as milk yield increased [42]. However, Kennelly [43] suggested that daily protein production might be unchanged due to the positive effect of supplemental fat on milk yield. In some cases, high dietary fat can decrease milk yield and protein value. This is possible because dietary fat supplementation negatively affects microbial fermentation and microbial protein yield, consequently decreasing the availability of amino acid supply for absorption [44].

Fat and FFA values in milk decreased significantly (p < 0.05) with the inclusion of extruded flaxseed or Salmate^®^ in the diets. Saturated short-chain FAs and saturated long-chain FAs contents in milk increased significantly (p < 0.05) with the supplementation of extruded flaxseed in the diet. More importantly, the omega-6: omega-3 ratio decreased from (8.06) in the control and (8.31) Salmate^®^ groups to (3.76) in the extruded flaxseed group (p < 0.05). Collectively, the results of this study reveal great importance for the dairy industry to improve milk quality. Milk quality is determined greatly by the content of n-3 FAs [45], which is fundamentally affected by the efficiency of transfer of FAs from animal feed to milk. The high-fat content of extruded flaxseed is 73.0% polyunsaturated, of which 78% belongs to the omega-3 family in the form of LA and ALA. The extruded flaxseed diet is 3 times richer in omega-3 than omega-6, making it superior compared to other oilseed sources such as cottonseed and soybean (Table-2).

### Ruminal disappearance and degradability characteristics

The ruminal degradability level (%) of DM, OM, CP, ADF, and NDF was determined at 3, 6, 9, 12, 24, and 48 h incubation time of feeding extruded flaxseed, Salmate^®^, and control diets (Table-3). The results showed that DM, OM, CP, and ADF disappearances or degradability (%) were lower (p < 0.05) in the extruded flaxseed and Salmate^®^ groups than in the control group. The degradation level of ADF was significantly (p < 0.05) the lowest of the extruded flaxseed groups, followed by Salmate^®^ and control groups, respectively. In addition, the degradability level of NDF was the lowest in the extruded flaxseed group and the highest level was in the Salmate^®^ group compared with the control group.

Degradability characteristics and effective degradability values are presented in Table-4. Such result might be attributed to heat processing of diets and retention time, which decreases in the extruded flaxseed and Salmate^®^ groups compared with the control group (Tables-6 and 7). Seifdavati and Taghizadeh [46] studied the effect of autoclaving legumes on protein degradation. They found no detrimental effect on protein quality due to heat processing, and the treated seeds could provide a source for microbial protein synthesis.

### Ruminal fermentation

The effects of extruded flaxseed and Salmate^®^ inclusion in the basal diet of lactating cows on ruminal pH, temperature, ammonia, and VFA concentrations in the ruminal fluid are shown in Table-5. There was no change (p > 0.05) in the ruminal pH of either the extruded flaxseed or Salmate^®^ groups compared with the control group, in addition to a significant decrease in the ruminal temperature of the extruded flaxseed group compared with the Salmate^®^ and control groups (Table-5). These results are in accordance with those of Benchaar *et al*. [36]. Changes in ruminal temperature are affected by heat stress [47] and might have consequences on vital processes such as ruminal fermentation [39], metabolic indices [48], and nutrient digestibility [7, 49].

The characteristics of rumen fermentation differed according to milk yield in the early stage of lactation. Sofyan *et al*. [50] suggested that high‐yield dairy cows seem to accommodate appropriately through rumen fermentation to negative energy balance in early lactation. The data in Table-5 show that ruminal fermentation as measured by NH_3_-N, total and individual VFAs, acetate, propionate, butyrate, valerate, and isovalerate molar proportions were not affected by the addition of extruded flaxseed and Salmate^®^ to the basal diet. The isobutyrate molar proportion was significantly higher (p < 0.05) in cows fed the extruded flaxseed (7.70) and control diets (7.32) than in those fed the Salmate^®^ diet (5.69). These results are in agreement with those of Benchaar *et al*. [36], who reported that ruminal total VFAs were not changed when cows were fed whole flaxseed or flax oil supplemented in the diet. These results confirm that rumen function was unaffected by the inclusion of extruded flaxseed or Salmate^®^ in the basal diet. The lack of effects on most ruminal fermentation characteristics of extruded flaxseed or Salmate^®^ supplements in the present study was due to the lower amounts of oil supplied compared with other studies [41, 51, 52] and variances between diet composition studies.

The butyrate molar proportion was unaffected by the extruded flaxseed and Salmate^®^ diet compared with the control diet. Benchaar *et al*. [36] stated that protozoa are butyrate producers and could be used as indicators of their activity in the rumen. Similar results were also obtained by Doreau *et al*. [53], who studied the effect of linseed lipid on ruminal metabolism and intestinal digestibility in cows and reported no change in protozoal number when cows’ diet was supplemented with either rolled flaxseed or a mixture of flax oil and flax meal. Santos *et al*. [54] stated that the inclusion of canola seeds had no effect on the pH, ammonia nitrogen, and VFA values in the rumen. They reported that extruded canola seeds might stimulate the efficiency of microbial protein synthesis and, thus, increase protein availability in the small intestine without compromising the total digestibility of protein.

### Kinetics of the solid and liquid passage rates

The results of the current investigation indicated that the inclusion of extruded flaxseed and Salmate^®^ in the basal diet resulted in a greater passage rate of digesta (solid phase) than the control diet, as reflected by the regression coefficients of −0.013, –0.012, and −0.007 for the extruded flaxseed, Salmate^®^, and control groups, respectively (Table-6). On the other hand, the passage rate of liquids out of the rumen expressed by the regression coefficient values for the extruded flaxseed (−0.028) and control (−0.021) groups significantly differed (p < 0.0001) from the regression coefficient of the Salmate^®^ group (−0.019), which indicates that cattle fed the extruded flaxseed and control diets had a greater liquid passage rate than those fed the Salmate^®^ diet (Table-7).

Knowledge of digesta passage is of great importance for predicting nutrient supply [55] using indigestible external markers. It has been indicated that animal species, diets, and ruminal temperature [47] might affect vital processes such as ruminal fermentation [39], metabolic indicators [48], and nutrient digestibility [7, 49].

Razzaghi *et al*. [56] reviewed the effects of the rate of passage of ruminal liquid (turnover) on ruminal fermentation. They stated that a faster liquid rate of passage from the rumen was associated with a decreased concentration of propionate and may contribute to improved milk fat percentage. The lower concentrations of ruminal NH_3_-N (10.19 mg/dL) and molar propionate (29.50) (Table-5) may indicate improved utilization of nitrogen due to the addition of extruded flaxseed to the basal diet. This, in turn, might explain the increase in milk yield of cows fed extruded flaxseed.

## Conclusion

The data from the current investigation show that the inclusion of extruded flaxseed (7.0%) and Salmate^®^ (25 g/head/day) in the diets of fistulated lactating Holstein cows did not have any notable negative effects on feed intake, ruminal fermentation parameters, and NH_3_-N. The positive effects of extruded flaxseed inclusion in the diet were observed in milk yield and composition by decreasing milk fat (%), increasing milk protein (%), and Omega-6: Omega-3 ratio.

## Authors’ Contributions

MA, TA, AE, and AAM: Conception of the study. TA: Conducted the study. MA and AE: Supervised the study. MA, TA, AE, and AAM: Extracted, verified, and analyzed the data and drafted and revised the manuscript. All authors have read, reviewed, and approved the final manuscript.
